# Secular trends in infant mortality by age-group and avoidable components in the State of São Paulo, 1996–2012

**DOI:** 10.1016/j.rppede.2016.03.009

**Published:** 2016

**Authors:** Kelsy Catherina Nema Areco, Tulio Konstantyner, José Augusto de Aguiar Carrazedo Taddei

**Affiliations:** a Departamento de Pediatria, Escola Paulista de Medicina, Universidade Federal de São Paulo (EPM-Unifesp), São Paulo, SP, Brazil

**Keywords:** Infant mortality, Health care (public health), Ecological studies, Time series studies

## Abstract

**Objective:**

To describe trends and composition of infant mortality rate in the State of São Paulo, from 1996 to 2012.

**Methods:**

An ecological study was conducted, based on official secondary data of births and infant deaths of residents in São Paulo, from 1996 to 2012. The infant mortality rate was calculated by the direct method and was analyzed by graphs and polynomial regression models for age groups (early neonatal, late neonatal and post-neonatal) and for groups of avoidable causes of death.

**Results:**

The mortality rate in the State of São Paulo tended to fall, ranging from 22.5 to 11.5 per thousand live births. Half of the infant deaths occurred in the early neonatal group. The proportion of avoidable infant deaths varied from 76.0 to 68.7%. The deaths which were avoidable by adequate attention to women during pregnancy and childbirth and newborn care accounted for 54% of infant deaths throughout the period.

**Conclusions:**

The mortality rate levels are still far from those in developed countries, which highlight the need to prioritize access and quality of healthcare services during pregnancy, childbirth and newborn care, especially in the first week of life, aiming at achieving standards of infant mortality similar to those of developed societies.

## Resumo

**Objetivo:**

Conhecer a tendência e a composição da taxa de mortalidade infantil no Estado de São Paulo de 1996 a 2012.

**Métodos:**

Estudo ecológico, baseado em dados secundários oficiais de nascimentos e óbitos infantis de residentes no Estado de São Paulo, de 1996 a 2012. A taxa de mortalidade infantil, calculada pelo método direto, foi analisada por meio de gráficos e modelos de regressão polinomial para os grupos etários (neonatal precoce, tardio e pós-neonatal) e para grupos de causas evitáveis de óbito**.**

**Resultados:**

A taxa de mortalidade infantil no Estado de São Paulo apresentou tendência de queda, variou de 22,5 a 11,5 por mil nascidos vivos. Metade dos óbitos infantis ocorreu no grupo etário neonatal precoce. A proporção de óbitos infantis evitáveis variou de 76,0% a 68,7%. As causas reduzíveis por adequada atenção à mulher na gestação e parto e ao recém-nascido representaram 54% dos óbitos infantis em todo o período.

**Conclusões:**

Os níveis da taxa de mortalidade infantil ainda estão distantes dos observados em países desenvolvidos, o que evidencia a necessidade de priorizar o acesso e a qualidade dos serviços assistenciais durante a gravidez, o parto e o período neonatal, principalmente na primeira semana de vida, para atingir padrões de mortalidade infantil próximos aos de sociedades desenvolvidas.

## Introduction

Infant mortality rate (IMR) reflects the living and health conditions of populations, estimates the risk of death among children under one year old, and expresses the inequities in different parts of the world, as can be seen in Africa and Europe, whose IMR in 2013 were 60 and 11 per 1000 live births (‰ LB), respectively.^1^^-^ 4

Reducing child mortality is one of the goals of the “eight Millennium Development Goals (MDGs)” established by the United Nations (UN) and agreed by Brazil, along with 190 nations in 2000. The Brazilian goal (IMR 15.7‰ LB by 2015) has already been met in 2011 (IMR 15.3‰ LB).^3^^,^ 5 However, due to its territorial extent the country has a large variability in IMR and difficulty in producing accurate estimates of this indicator.^6^^,^ 7 The highest IMR are in the North and Northeast and the lowest in the South and Southeast.^5^^,^ 7 For example, in 2008-2010, the IMR corrected by active search of vital statistics was 11.2‰ LB in Santa Catarina and 28.7‰ LB in Amapá.^7^

São Paulo State, the country's main socioeconomic region, with 18.2% of Brazilian infant deaths, had an IMR of 11.6‰ LB in 2011 and ranks third among states. When compared to the Latin America countries, the State of São Paulo only has rates higher than those of Cuba and Chile, which showed IMR below 10‰ LB. Although within the agreed target, the IMR of São Paulo is far from those of developed countries, which are not higher than 5‰ LB, showing that there is still considerable room for improvement.^3^^,^ 5 ^,^ 8 ^,^ 9

In Brazil, in the 1980s, the main causes that contributed to the reduction of infant mortality in Brazil belonged to the group of preventable diseases through proper sanitation, and half of the deaths occurred in the post-neonatal period.^9^^,^ 10 The majority of infant deaths occur in the neonatal period and are associated with both the quality and access to health services that provide prenatal, childbirth, and newborncare.^1^^-^ 3 ^,^ 11 Despite this change in the scenario of infant deaths over time, the preventable conditions as determinants of infant mortality predominate.

In 1977, the analysis of preventable deaths was proposed as a health care quality indicator, and defined as those that should not occur if health centers were effective.^2^^,^ 12 From this concept, researchers in different countries have developed preventability criteria to identify these preventable deaths. The first Brazilian list for classification of avoidable causes of infant deaths was proposed in 2000 by the Fundação Sistema Estadual de Análise de Dados do Governo do Estado de São Paulo (SEADE - a state system of data analysis), according to which the avoidable deaths are those that can be prevented, regardless of the availability of local resources, technologies, existing procedures or treatments. In 2007, the current Brazilian list applicable to children under five years old was developed, based on existing classifications, concepts of preventable deaths related to actions of health services and technology available in the Unified Health System (“Sistema Único de Saúde” - SUS).^1^^,^ 2 ^,^ 13

Knowledge of the infant mortality trend in São Paulo State helps to map the evolution and current state of this population health status and provides important information to local administrators who design reduction and prevention strategies for infant mortality. In this context, the aim of this study is to describe the secular trend of infant mortality rate in the State of São Paulo by age group and group of avoidable causes through actions of health services.

## Method

An ecological study based on secondary data of births and deaths, collected by the Ministry of Health (MH) through the Live Birth Information System (“Sistema de Informação sobre Nascidos Vivos” - SINASC) and Mortality (“Sistema de Informações de Mortalidade” - SIM), which is available in structured digital format.^14^

We included all birth events (live births) and infant mortality (under one year old), referring to the children of mothers living in São Paulo, occurred from 01/01/1996 to 12/31/2012. These deaths were classified according to lifetime (age group), preventability, and group of avoidable causes.

IMR, expressed in number of deaths per thousand live births (‰ LB), was calculated by the direct method,^15^ year by year, for categories of dimensions in study (age, death preventability, and group of preventable causes).

Regarding age, deaths were grouped into three categories: early neonatal (0-6 days), late neonatal (7-27 days), and post-neonatal (28-364 days).^16^

Deaths were classified according to the preventability from the basic cause of death, according to the Brazilian list of causes of preventable deaths in children under five years old.^2^^,^ 17

IMR trend was assessed using graphs and polynomial regression models, adjusted for each category of interest (general, age group, and group/subgroup of causes). IMR was the dependent variable (*Y*) and the study years were the only independent variable (*X*). The year 1996 was considered as the “year zero” and a significance level of *α*=0.05 was considered for all tests performed. First, a significance test was performed for linear model, with an investigation of the coefficient of determination (*R* ^2^) and the violation of adherence assumptions for dependent variable (*Y*) and residuals to the normal distribution, homoscedasticity, and absence of serial correlation residuals. This process was repeated with the statistical package STATA/IC12.1, increasing the polynomial order (2nd, 3rd, 4th, 5th order) up to a model without assumption violation. The coefficient of determination (*R* ^2^) of each model was used as a measure of fitting adequacy, considering those closest to the unit as the best fitted.^18^^,^ 19

Total and mean variations were calculated in IMR units (‰ LB) and respective percentages (%) for both observed and estimated values. The percentage changes were presented to the nearest 0.1 (1 decimal place), but were calculated to six decimal places.

The mean number of deaths corresponding to the variation of 0.1‰ LB in IMR was calculated based on the mean number of live births.

This study was approved by the Institutional Review Board of the Federal University of São Paulo, under the Opinion number 664.976.

## Results

**Figure 1:**
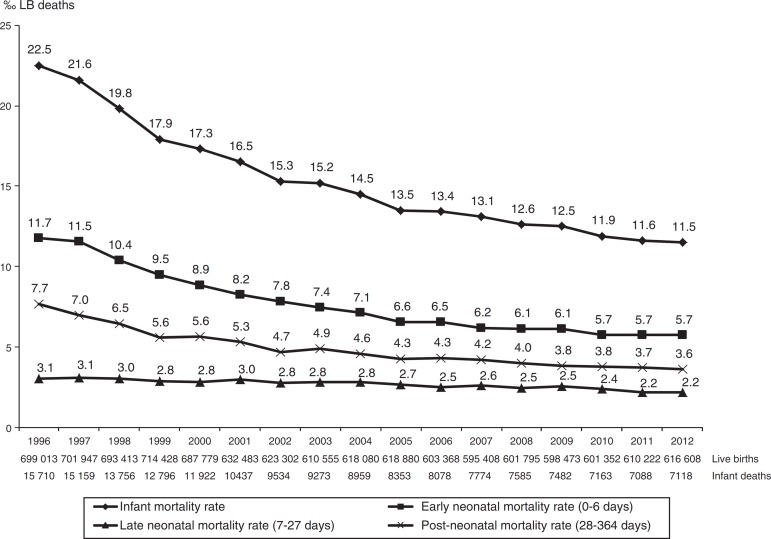
Infant mortality rate according to age. State of São Paulo, 1996-2012.

From 1996 to 2012, 10,827,106 live births and 168,187 infant deaths (under one year old) were recorded, children of mothers living in the State of São Paulo, and 99.8% of deaths occurred within the state itself (Fig. 1).

The infant mortality rate ranged from 22.5 to 11.5 deaths per thousand live births (‰ LB), with a percentage decrease of 48.7% and average estimated reduction of 0.65‰ LB annually. On average, the one-tenth (0.1) variation in IMR corresponded to the effect magnitude of 63.7 infant deaths per thousand live births. Considering the average annual number of live births during the study period (1996-2012), which was 636,888, the annual estimated drop of 0.65‰ LB in IMR corresponded to the mean annual reduction of 414 deaths (Fig. 1 and Table 1).

**Table 1:** IMR trend analysis according to age and group of causes of death. State of São Paulo, 1996-2012.

	IMR observed variation			IMR estimated variation^a^		*R* ^2^
	Total	Annual average		Total	Annual average	
**IMR**	↓10.93‰	↓0.68‰		↓10.46‰	↓0.65‰	0.99
	(48.7%)	(3.1%)		(46.9%)	(2.9%)	

*IMR by age*						
Early neonatal	↓6.00‰	↓0.38‰		↓6.17‰	↓0.39‰	0.99
	(51.3%)	(3.2%)		(51.9%)	(3.2%)	
Late neonatal	↓0.86‰	↓0.05‰		↓0.85‰	↓0.05‰	0.92
	(28.2%)	(1.8%)		(27.3%)	(1.7%)	
Post-neonatal	↓4.07‰	↓0.25‰		↓3.58‰	↓0.22‰	0.97
	(52.9%)	(3.3%)		(48.6%)	(3.4%)	

*IMR by group of causes of death*						
Ill-defined causes	↓0.71‰	↓0.04‰		↓0.72‰	↓0.04‰	0.96
	(65.8%)	(4.1%)		(67.1%)	(4.2%)	
Other causes (not clearly preventable)	↓1.03‰	↓0.06‰		↓1.10‰	↓0.07‰	0.88
	(24.0%)	(1.5%)		(26.0%)	(1.6%)	

**Preventable causes**	↓9.19‰	↓0.57‰		↓9.26‰	↓0.58‰	0.99
	(53.7%)	(3.4%)		(53.7%)	(3.4%)	
*Reducible through proper care for women during pregnancy, childbirth, and newborn care*	↓5.63‰	↓0.35‰		↓6.08‰	↓0.38‰	0.99
	(46.5%)	(2.9%)		(49.2%)	(3.1%)	
*Reducible through proper care for women during pregnancy*	↓1.71‰	↓0.11‰		↓1.54‰	↓0.10‰	0.92
	(36.0%)	(2.3%)		(32.2%)	(2.0%)	
*Reducible through proper care for women during childbirth*	↓1.40‰	↓0.09‰		↓1.50‰	↓0.09‰	0.97
	(57.8%)	(3.6%)		(60.0%)	(3.8%)	
*Reducible through proper care for the newborn*	↓2.52‰	↓0.16‰		↓2.67‰	↓0.17‰	0.98
	(50.9%)	(3.2%)		(52.2%)	(3.2%)	
*Reducible through proper diagnostic and treatment actions, health promotion linked to attention and immunization measures*	↓3.57‰	↓0.22‰		↓3.55‰	↓0.22‰	0.99
	(71.2%)	(4.4%)		(71.8%)	(4.5%)	
*Reducible through proper diagnostic and treatment actions*	↓2.32‰	↓0.15‰		↓2.28‰	↓0.14‰	0.99
	(72.7%)	(4.5%)		(72.6%)	(4.6%)	
*Reducible through health promotion linked to care actions*	↓1.25‰	↓0.08‰		↓1.17‰	↓0.07‰	0.97
	(70.2%)	(4.4%)		(68.2%)	(4.3%)	
*Reducible through immunizing measures*	↑0.01‰	0.00‰		↓0.01‰	0.00‰	0.71
	(60.1%)	-		(28.3%)	-	

a All estimates were generated from polynomial regression models statistically significant (*p*<0.001); *R* ^2^, coefficient of determination.

The early neonatal IMR ranged from 11.7‰ to 5.7‰ LB, down 51.3%; late neonatal ranged from 3.1‰ to 2.2‰ LB, down 28.2%; and post-neonatal ranged from 7.7‰ to 3.6‰ LB, down 52.9%. Regarding the total number of deaths analyzed, early neonatal accounted for 50.5%, late neonatal for 17.4%, and post-neonatal for 32.1% of infant deaths. The contribution of early neonatal deaths to IMR ranged from 52% (11.7‰-22.5‰ LB) in 1996 to 49.6% (5.7‰-11.5‰ LB) in 2012 (Fig. 1).

In all age groups, the observed and estimated values show that IMR tended to drop in this period, with annual reduction estimated at 0.39‰ LB for early neonatal; 0.22‰ LB for post-neonatal, and 0.05‰ LB for late neonatal deaths (Fig. 1 and Table 1).

**Figure 2:**
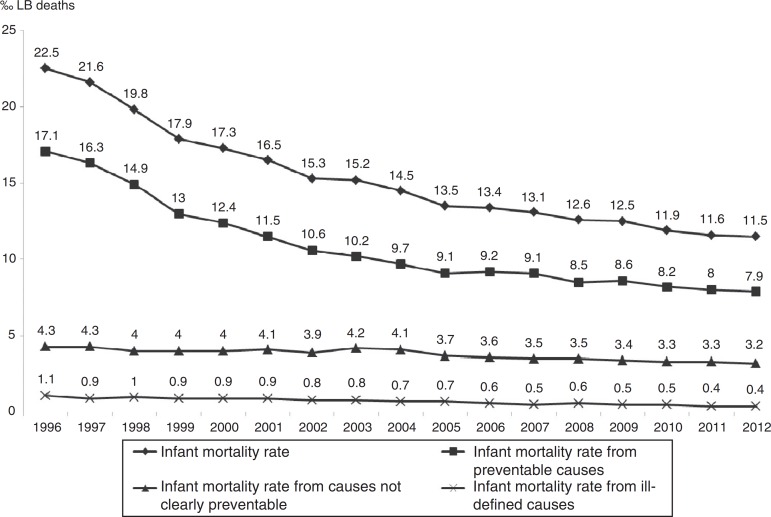
Infant mortality rate according to death preventability. State of São Paulo, 1996-2012.

Regarding death preventability, the observed IMR from preventable causes ranged from 17.1‰ LB to 7.9‰ LB, down 53.7%; IMR from causes not clearly preventable ranged from 4.3‰ LB to 3.2‰ LB, down 24%; and IMR from ill-defined causes ranged from 1.1‰ LB to 0.4‰ LB, down 65.8%. Deaths from preventable causes accounted for the largest share of infant deaths throughout the period, ranging from 76% (1996) to 69% (2012). In the three groups analyzed, there was annual reduction estimated at 0.04‰ LB for ill-defined causes, 0.07‰ LB for causes not clearly preventable, and 0.58‰ LB for preventable causes (Fig. 2 and Table 1).

**Figure 3:**
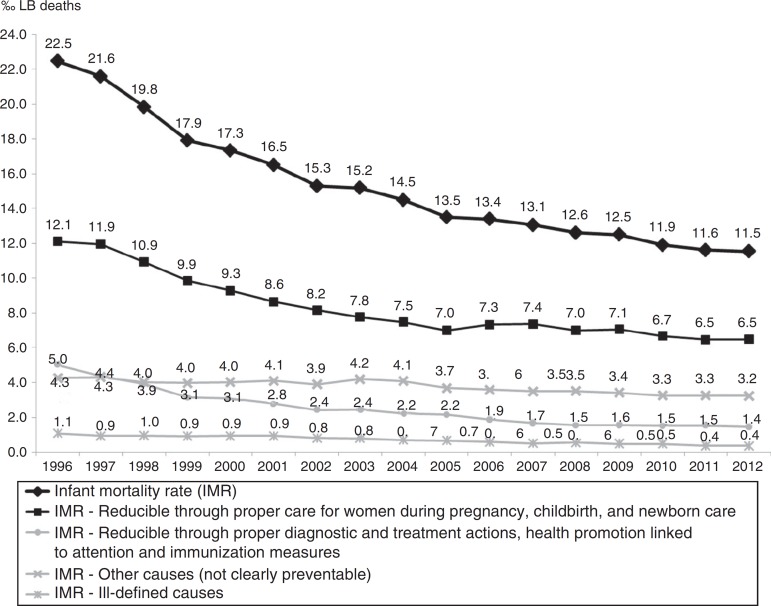
Infant mortality rate by group of causes. State of São Paulo, 1996-2012.

The causes of death “Reducible through proper care for women during pregnancy, childbirth, and newborn care” dropped by 46.5%, ranging from 12.1‰ LB to 6.5‰ LB, with estimated annual reduction of 0.38‰ LB. Infant deaths from these causes accounted for 54.3% of the total in the period. The downward trend was also observed in subgroups, with reductions estimated at 0.10‰ LB for causes related to women during pregnancy, 0.09‰ LB for causes related to women during childbirth, and 0.17‰ LB for causes related to newborn care (Fig. 3 and Table 1).

The IMR reduction observed in all causes “Reducible through proper diagnostic and treatment actions, health promotion linked to attention and immunization measures” was 71.2%, ranging from 5‰ LB in 1996 to 1.4‰ LB in 2012, with annual estimated reduction of 0.22‰ LB. The average annual reduction was estimated at 0.14‰ LB for the causes “Reducible through proper diagnostic and treatment actions” and 0.07‰ LB for the causes “Reducible through health promotion actions linked to attention actions”(Fig. 3 and Table 1).

The IMR for causes “Reducible through immunization measures” had an observed average of 0.02‰ LB and an observed and estimated average annual variation close to zero (0.00‰ LB).

## Discussion

Between 1996 and 2012, the IMR in São Paulo dropped by approximately 50% and showed a downward trend, significantly more pronounced in the first half of this period, with evasion of only 0.2% of deaths. From 1997 to 2006, the São Paulo IMR reduced by 38%, a greater reduction than that for Brazil (35.2%).^1^ This reduction in national IMR seems to result from improvement in health services, as the performance of other social indicators at that time were not as good.^1^

With the challenge of achieving the national and internationally agreed goals for the proposed IMR levels for 2015, it became necessary to monitor this indicator in different administrative levels to assess the impact of the decentralized, but nationally articulated, health actions.^6^ Consequently, there was an increased demand for more accurate estimates of IMR, which depend on good coverage of vital records and regularity in sending information to SINASC and SIM systems.^6^^,^ 7 ^,^ 15

This study estimated the annual IMR for the State of São Paulo using the direct method, without need for using other procedures or correction factor for the whole period because vital information was considered complete in this state, according to the Interagency Network of Health Information (“Rede Interagencial de Informações para a Saúde” - RIPSA) criteria, as well as in Rio de Janeiro, Rio Grande do Sul, and Mato Grosso do Sul.^5^^,^ 6 ^,^ 15

The age composition of infant deaths assessed in the State of São Paulo was similar to that in Brazil, with prevalence of neonatal group.^1^^,^ 3 ^,^ 20 In 2011, the São Paulo neonatal IMR was lower than that of Brazil (11.1‰ LB) and similar to that of the Southeast region (8.0‰ LB).^3^ Neonatal mortality is a result of the quality of prenatal consultations care for women during pregnancy, childbirth, and newborn care.^3^^,^ 21 ^,^ 22 Many neonatal deaths can be avoided, even in premature with very low birth weight.^22^

Accounting for about half of infant deaths, the early neonatal group also stood out with the highest average annual reduction in mortality rate during the 17 years studied. Therefore, variations in mortality in this age group have great impact on child mortality. Despite the downward trend, the early neonatal mortality rate in 2012 (5.7‰ LB) was three times that observed in 2010 in all 28 countries of the European Union.^23^

Among the preventable causes of neonatal deaths, we highlight perinatal asphyxia. It represented 27.1% of São Paulo neonatal deaths in 1996 and 17.4% in 2012. This reduction has been attributed to the organization of perinatal care in the various levels of action, including those aimed at pregnant women, childbirth, and newborn. Note worthily, the neonatal resuscitation program established in 1996 by the Brazilian Society of Pediatrics, which aims to reduce preventable child deaths and the consequent neurological sequelae of perinatal asphyxia, and the regionalized strategies, such as the program *Mãe Paulistana do Município de São Paulo* (“Mothers from the city of São Paulo”).^24^^-^ 26

From 1996 to 2012, the proportion of preventable infant deaths ranged from 76% to 69% in São Paulo. In Brazil, this proportion ranged from 71.4% in 1997 to 69.4% in 2006.^1^ Although the time intervals and the quality of information^6^^,^ 7 ^,^ 17 on the cause of death will not allow us the direct comparison of values, these data show that since 1996, most infant deaths were avoidable, both in São Paulo and throughout Brazil.

The group of causes “Reducible through proper care for women during pregnancy, childbirth, and newborn care”’ accounted for more than half of infant deaths analyzed. The IMR reduction in this group may be associated with increased access to health services and increased health care quality provided to pregnant women and newborns.^21^

The stability at low levels of mortality in the group of preventable causes through immunizing measures reflected the high rates of the National Immunization Program vaccination coverage.^27^ The average vaccination coverage in children under one year old in the State of São Paulo, between 2000 and 2012, was greater than 97%, including meningococcal conjugate C introduced in 2011. The human rotavirus vaccine and 10-valent pneumococcal conjugate vaccine, introduced in 2006 and 2010, respectively, have the lower coverage, reaching over 86% in 2011.^28^

The period under study, with IMR calculated year by year, was enough to perform the inferential analysis of this indicator trend in all categories evaluated. Although the trend could be observed by visual inspection of the population values, we chose to follow the recommended method for trend analysis in time series, which consisted of the analysis adjusted by polynomial model for each category. By using the model-estimated values, we avoided that the possible fluctuations in values, occurring at random because they are dependent on the total number of reported deaths and births, interfere in the time series trend analysis.^18^ The coefficients of determination (*R* ^2^) close to the unit, as well as the observed values close to the estimated values, indicated good quality adjustment data. However, the downward trend should not be rigidly extrapolated to future periods, because conceptually, the lower the rate of any biological event, the less the chances of reducing it.

To classify death preventability, the basic cause reported in death records were used in our study, considering that it was de illness that started the chain of events leading to death.^15^ It is therefore the most important diagnostic for epidemiological purposes, in which most studies of causes of death in Brazil are based. The higher the proportion of deaths from ill-defined causes, the worse the quality of information on the underlying cause of death. In the present study, we found a low proportion of deaths from ill-defined causes, ranging from 4.8% to 3.2%, which enabled this analysis.^17^^,^ 29

The regional differences within the State of São Paulo, both in the access to health services and technologies available, were not considered in this study. It was assumed that the causes of child death would be equally preventable in 675 municipalities of the state, the used list assumes the existence of SUS in all regions with the same availability of resources and health care technologies.^2^

By classifying infant deaths to study the trend of IMR from preventable causes, we consider that the different historical moments do not differ in technology and health practices. In this study, the IMR of preventable deaths in the 17 years analyzed may be overestimated in the years before its creation (2007) and underestimated in subsequent years, it is assumed that more causes of death become preventable with the progressive improvement in the technologies used in the health system.^2^^,^ 13

The list adopted in this study considers as preventable causes of death the morbidities related to gestational age extremes (short or long) and birth weight (low or high), which are birth conditions commonly associated with risk of death.^2^^,^ 22

The analyzed deaths refer to the users of the public (SUS) and supplementary health system of the State of São Paulo, but it was not possible to quantify the contribution of each system in infant mortality, as this information is not included in the SIM. One can have an idea of the supplementary health services participation in SUS services from the coverage rate of these services, which ranged from 38.2% in 2000 to 45.3% in 2014. Under the assumption that the same causes of preventable deaths in the SUS would also be preventable in the supplementary health system, it should be considered that the analysis of IMR from preventable causes (by SUS interventions) in this study contributes to the evaluation of the State of São Paulo health system performance as a whole and highlights the kind of health care that deserve priority in order to reduce infant mortality, both in the SUS and in the supplementary health system.^1^^,^ 2 ^,^ 30

In this context, we conclude that the infant mortality rate in the State of São Paulo remained in decline between 1996 and 2012 and had as main components early neonatal deaths and preventable causes, particularly those related to pregnancy, childbirth, and neonatal care. However, the rates for the end of the study period are still far from those seen in developed countries. These evidences indicate an improvement in the health system, but also the need to improve and expand health care services to promote health and prevent deaths in the first year of life, in order to achieve standards or mortality profiles close to those of developed societies.
